# Xuesaitong promotes myocardial angiogenesis in myocardial infarction mice by inhibiting MiR-3158-3p targeting Nur77

**DOI:** 10.18632/aging.204671

**Published:** 2023-04-20

**Authors:** Jiangquan Liao, Mingjing Shao, Yan Wang, Peng Yang, Dongliang Fu, Mengru Liu, Tong Gao, Kangkang Wei, Xianlun Li, Jinhang Du

**Affiliations:** 1 National Integrated Traditional and Western Medicine Center for Cardiovascular Disease, China–Japan Friendship Hospital, Beijing, China; 2 Department of Cardiology, Beijing Tsinghua Changgung Hospital, Medical Center, Tsinghua University, Beijing, China; 3 Graduate School, Beijing University of Chinese Medicine, Beijing, China

**Keywords:** Xuesaitong, miR-3158-3p, myocardial infarction, angiogenesis

## Abstract

This study aims to investigate the regulatory effect of Xuesaitong (XST) and miR-3158-3p on angiogenesis. All mice were randomly assigned into Sham group, Model group, XST group, XST + miR-3158-3P-overexpression (miRNA-OE) group. XST was found to increase the left ventricular anterior wall thickness at end diastole and end systole (LVAWd and LVAWs), left ventricular internal dimension at end diastole and end systole (LVIDd and LVIDs), fractional shortening (FS), and ejection fraction (EF) and decrease the proportion of fibrotic areas in mice. In contrast to those in Sham group, the protein expressions of Nur77, p-PI3K, HIF-1α, VEGFs, COX-2 in the heart tissues of mice in Model group were elevated and further increased after XST treatment in comparison with those in Model group. Nur77-/- mice were utilized. It was found that XST enhanced cell viability through a methyl thiazolyl tetrazolium assay and facilitated angiogenesis in each group, as assessed by a catheter formation assay. Specifically, XST was shown to promote the formation of blood vessels. Moreover, the protein expression levels of Associated proteins in the heart tissues of Nur77-/- mice were dramatically reduced in mice in Model and XST group compared with those in WT mice. Additionally, the above-mentioned protein expressions in the heart tissues of Nur77-/- mice did not change significantly in mice in Model + miRNA-OE + XST group compared with those in WT mice, suggesting that miR-3158-3p can specifically inhibit the expression of Nur77. In conclusion, XST inhibits miR-3158-3p targeting Nur77 to facilitate myocardial angiogenesis in mice with myocardial infarction.

## INTRODUCTION

Vascular endothelial cells are barriers between blood and tissues. They secrete various vasoactive substances, which are crucial for maintaining the integrity of the vascular wall, regulating vascular tension, activating and aggregating platelets, and remodeling the vascular wall [1]. Vascular endothelial injury and dysfunction are the initial links of numerous cardiovascular diseases [2]. Besides, vascular endothelial cells release and synthesize a variety of vasoactive factors to exert vital regulatory effects on vascular homeostasis [3]. Endothelial dysfunction results from the unbalance between vasoactive substances and cytokines synthesized and secreted by the injured endothelium [4].

Xuesaitong (XST) mainly comprises Panax notoginseng saponin (PNS). PNS has a hemostatic effect and can enrich the blood, which is the main active ingredient of Panax notoginseng, a classic drug for promoting blood circulation and removing blood stasis [5]. Panax notoginseng can facilitate cell division, growth, and proliferation [6], and plays various roles in the vascular system [7], nervous system [8], blood system [2], and inflammatory responses [7], but it has been mainly applied in cardiovascular and cerebrovascular diseases [9]. Previous experiments have shown that XST is capable of reducing platelet adhesion and aggregation, improving microcirculation, enhancing the activity of oxidized human umbilical vein endothelial cells (HUVECs), and up-regulating the expression of the vascular endothelial growth factor (VEGF) signaling pathway, so as to facilitate angiogenesis [5]. Angiogenesis is defined as the formation of new blood vessels in the human body, and it is vital for various biological processes. In addition, angiogenesis is crucial for treating numerous diseases, including stroke, myocardial infarction, and cardiovascular diseases. The deficiency of blood vessels in active metabolic tissues may impede injury repair or other basic functions. It is widely recognized that myocardial infarction often occurs with angiogenesis and collateral vascular circulation, which help mitigate cardiac fibrosis [10, 11]. Actually, the blood supply can be increased, and ischemic brain function can be improved by impelling the angiogenesis in the ischemic brain and increasing the number of circulating collateral vessels.

Furthermore, micro ribonucleic acids (miRNAs) are natural short non-coding RNAs that can inhibit the expression of specific genes encoding target proteins after transcription [12]. Their vital roles in the biology of blood vessels and endothelial cells have been verified by new evidence in recent years [13], but how XST modulates the angiogenesis of endothelial cells through miRNAs remains unclear. In this study, therefore, the regulatory role of XST in the angiogenesis of endothelial cells was first determined, and the specific mechanism by which XST affects endothelial cell function by regulating miR-3158-3p was then explored, so as to provide a theoretical basis for applying XST in the clinical treatment of vascular diseases such as myocardial infarction.

## MATERIALS AND METHODS

### Bioinformatics analysis

Through searching for the data set related to myocardial infarction in the Gene Expression Omnibus (GEO) database (https://www.ncbi.nlm.nih.gov/gds/), GSE83500, the data set of myocardial infarction-related mRNA gene expression, and GSE76604, the data set of myocardial infarction-related miRNA sequencing, were found and downloaded. Additionally, the quantile standardization and differential gene analysis of RNA-seq data were carried out using R language limma software package (/logFC/>1, P<0.05). Then ggplot2 software package was utilized to plot the visualized volcano map of grouped differentially expressed genes (DEGs) in GSE83500 in R software, and the cluster analysis heat map of DEGs was drawn by R software package pheatmap. Likewise, the visualized volcano map and cluster analysis heat map of grouped DEGs in GSE76604 were plotted.

### Functional enrichment analysis

DEGs in GSE83500 were subjected to Gene Ontology (GO) and Kyoto Encyclopedia of Genes and Genomes (KEGG) enrichment analyses. Besides, DEGs in biological processes, cell compositions, and molecular functions were analyzed using the online database tool DAVID (https://david.ncifcrf.gov) to integrate GO terms and create a biological process network of DEGs. Moreover, the GO and KEGG pathway enrichment analysis diagrams of DEGs were plotted using GOplot and ggplot2 packages in the R language environment.

### Gene set enrichment analysis (GSEA)

GSEA was performed for all genes using the GSEA tool (http://www.gsea-msigdb.org/), and its pathway diagram was drawn.

### Protein-protein interaction (PPI) network analysis of messenger RNA (mRNA) DEGs and screening of target genes

The screening of interacting proteins with a combined score>0.9 was carried out by entering DEGs into the online tool STRING. Then the obtained PPI results were imported into the Cytoscape software, and the target genes with a score <10 were obtained through a calculation based on the degree algorithm with Cytohubba plug-in.

### Prediction of miRNA target genes

The online tools starBase and targetScan were utilized to predict the candidate target genes of miRNAs. The Venn diagrams of these candidate genes and GSE76604 DEGs were plotted using the VennDiagram package to predict the target genes. Finally, the binding site between mRNAs and miRNAs was mapped according to gene prediction results.

### Animal modeling and grouping

All the mice were randomly divided into six groups, namely, Control group, sham operation group (Sham group), Model group, XST group (intragastric administration with XST immediately after operation), and XST + miR-3158-3p-overexpression (miRNA-OE) group (injected with miR-3158-3p mimic via the tail vein). The optimal dose was selected according to the results of cardiac ultrasonography. Next, the mouse model of myocardial infarction was established according to the literature [14]. Specifically, the mice were intraperitoneally injected with pentobarbital sodium hydrochloride (30 mg/kg) for anesthetization. Then ventilator-assisted breathing of mice was performed through orotracheal intubation, followed by thoracotomy and coronary artery ligation (the ligation site was about 1.0-1.5 mm from the lower edge of the left atrial appendage). After body surface ECG monitoring results indicated successful modeling, the chest wall was sutured. Subsequently, the physiological state of the mice was observed to be stable, and penicillin was injected to prevent infection after operation. In Sham group, only thoracotomy was performed, with no coronary artery ligation. Furthermore, the mice treated with XST (40 mg/kg) received intragastric administration immediately after operation, and some of them were injected with miR-3158-3p mimic via the tail vein. After that, the mice were further grouped into Sham group, Model group, Model + XST group, and Model + miRNA overexpression (OE) + XST group.

### Cardiac ultrasonography

After anesthesia by inhalation with 2.0% isoflurane, the mice were fixed in the supine position on a heating plate at a constant temperature (37° C). Their breasts and upper abdomen underwent depilation to expose their skin fully. After that, they were examined by Vevo2100 small animal ultrasonic apparatus using an MS-550D probe. Later, the long-axis section of the sternum was taken, and the short-axis section of the left ventricle could be detected by rotating the probe clockwise at 90° on the basis of the long-axis section of the left ventricle. Afterward, the movement of the left ventricle was recorded by M-mode, followed by measurement of the left ventricular anterior wall thickness at end diastole and end systole (LVAWd and LVAWs), left ventricular internal dimension at end diastole and end systole (LVIDd and LVIDs), fractional shortening (FS), and ejection fraction (EF).

### Morphological observation

Paraffin-embedded sections were deparaffinized and added with Regaud or Weigert hematoxylin for 5-10 min of nucleus staining. After washing with water, the sections were stained by Masson ponceau acid reddening solution for 5-10 min, rinsed with 2% glacial acetic acid aqueous solution for a while, and differentiated using 1% phosphomolybdic acid aqueous solution for 3-5 min. After that, the sections were directly dyed using an aniline blue or light green solution for 5 min and soaked in 0.2% glacial acetic acid aqueous solution for a while. Subsequently, they were transparentized with 95% alcohol, absolute alcohol, and xylene and then sealed with neutral balsam. Following drying, the sections were observed and photographed under a microscope. Finally, the fibrotic area of cardiac lesions was analyzed by Image Pro-Plus-6 software (Media Cybernatics, Rockville, MD, USA).

### Methyl thiazolyl tetrazolium (MTT) assay

A single cell suspension was prepared using a culture medium containing 10% fetal bovine serum. Cells were seeded into a 96-well plate (200 μL/well) at 1×10^5^ cells/well and incubated under the same general culture conditions for three days. Later, 20 μL of MTT solution (5 mg/mL in PBS) was added per well. The plate was then incubated for an additional 4 h, after which the culture was stopped, and the in-well culture supernatant was carefully aspirated. The suspension cells were then centrifuged, and the in-well culture supernatant was discarded. Next, 150 μL of DMSO was added per well, and the plate was shaken for 10 min to dissolve the crystals fully. The enzyme-linked immunosorbent assay (ELISA) kit was used to measure the optical density (OD) value at the wavelength of 490 nm. The results were recorded, with the time as the abscissa and the OD value as the ordinate.

### Extraction and culture of cardiomyocytes

Cardiomyocytes were isolated from the heart tissue of WT suckling mice and nerve growth factor-induced gene B knockout (Nur77^-/-^) suckling mice aged 1-4 days old. First, the chest skin was disinfected with 75% ethanol, and the heart was removed from the mice and placed into a flat M containing D-Hanks solution. The atrium and ventricles were then cut, and the heart was rinsed three times with D-Hanks solution to remove the residual blood. Later, the heart tissue was cut into small fragments (1 mm^3^) and transferred to a centrifuge tube containing digestive juice (5 mL). The tissue was then digested for 5 min at 37° C, and the supernatant was discarded after natural precipitation. This process was repeated for 20 min with a fresh digestion solution. After blowing for 1 min with a pipette, the undigested heart fragments were aspirated into another centrifuge tube and mixed with a pre-cooled culture medium (2 mL) to terminate the digestion process. The resulting solution was centrifugated at 1000 rpm for 5 min to discard the supernatant, washed with 2 mL of D-Hanks solution, and resuspended in 2 mL of culture medium. After blowing with a pipette, a cell suspension was prepared and incubated in an incubator with 5% CO_2_ at 37° C.

### Co-culture of cells

Primary cardiomyocytes extracted from WT suckling mice and Nur77^-/-^ suckling mice were inoculated into the Transwell chamber and cultured for 24 h. C166 mouse vascular endothelial cells were seeded into cell culture plates and cultured for 24 h. After overnight incubation, the culture medium in the Transwell chamber and plate was removed, and a new culture medium was added to the lower compartment of the Transwell chamber. Then the chamber was placed into the cell culture plate for 48-72 h of incubation. After that, the Transwell chamber was removed, and the culture medium was aspirated from the cell culture plate. The cells were then washed twice with PBS and fixed with 4% paraformaldehyde.

### Tube formation assay

A matrix gel was coated onto a 24-well plate (BD Biosciences, Franklin Lakes, NJ, USA). The unpolymerized matrix was placed in wells (300 μL/well) and polymerized for 1 h at room temperature. C166 cells were then seeded onto the polymerized matrix at 5×10^4^ cells/well in 500 μL of the medium. The angiogenic stimulators, VEGF-A (Shanghai BestMart Technology Co., Ltd., China) and basic fibroblast growth factors (bFGFs) (Shanghai Bioart Technology Co., Ltd., China), were added to the medium at a concentration of 10 ng/mL each, respectively. The cells were then incubated with 5% CO_2_ for 18 h at 37° C. After incubation, the tumor formation images were acquired using an inverted phase-contrast optical microscope equipped with a microscope camera (Q Imaging, Surrey, BC, Canada) (Olympus Corporation, Tokyo, Japan).

### Western blotting

A protein extraction kit was utilized to extract cell proteins and determine the protein concentration. Next, 50 μg of proteins received sodium dodecyl sulfate-polyacrylamide gel electrophoresis (SDS-PAGE), and the proteins on the gel were transferred onto a nitrocellulose membrane by point transfer. Later, the proteins were sealed with 3.0% skim milk powder, and incubated with primary antibodies against Nur77, phosphorylated phosphatidylin-ositol-3-kinase (p-PI3K), total PI3K (t-PI3K), phosphorylated mammalian target of rapamycin (mTOR) (p-mTOR), total mTOR (t-mTOR), hypoxia-inducible factor 1α (HIF-1α), VEGFs, cyclooxygenase-2 (COX-2) and glyceraldehyde 3-phosphate dehydrogenase (GAPDH) diluted at 1: 10000 at 4° C overnight. After membrane washing, the proteins were incubated with horseradish peroxidase-labeled goat anti-rabbit secondary antibody (1:10000) at room temperature for 2 h. At last, images were collected after chemiluminescence, and their gray scales were analyzed using Quantity One analysis software.

### Statistical analysis

Bioinformatics analysis was conducted by R v3.6.1 software package DEseq2 and ggpubr statistical software package. Wald test was adopted for DEG analysis, and the rank-sum test was employed for cytokine comparison between two groups. Other indexes were expressed as mean ± standard deviation (*x*±s). Besides, the one-way analysis of variance of the differences in each index was carried out using SPSS 22.0 software, and pairwise comparison was performed via LSD test. P<0.05 represented that the difference was statistically significant.

## RESULTS

### Screening results of DEGs

**Figure 1:**
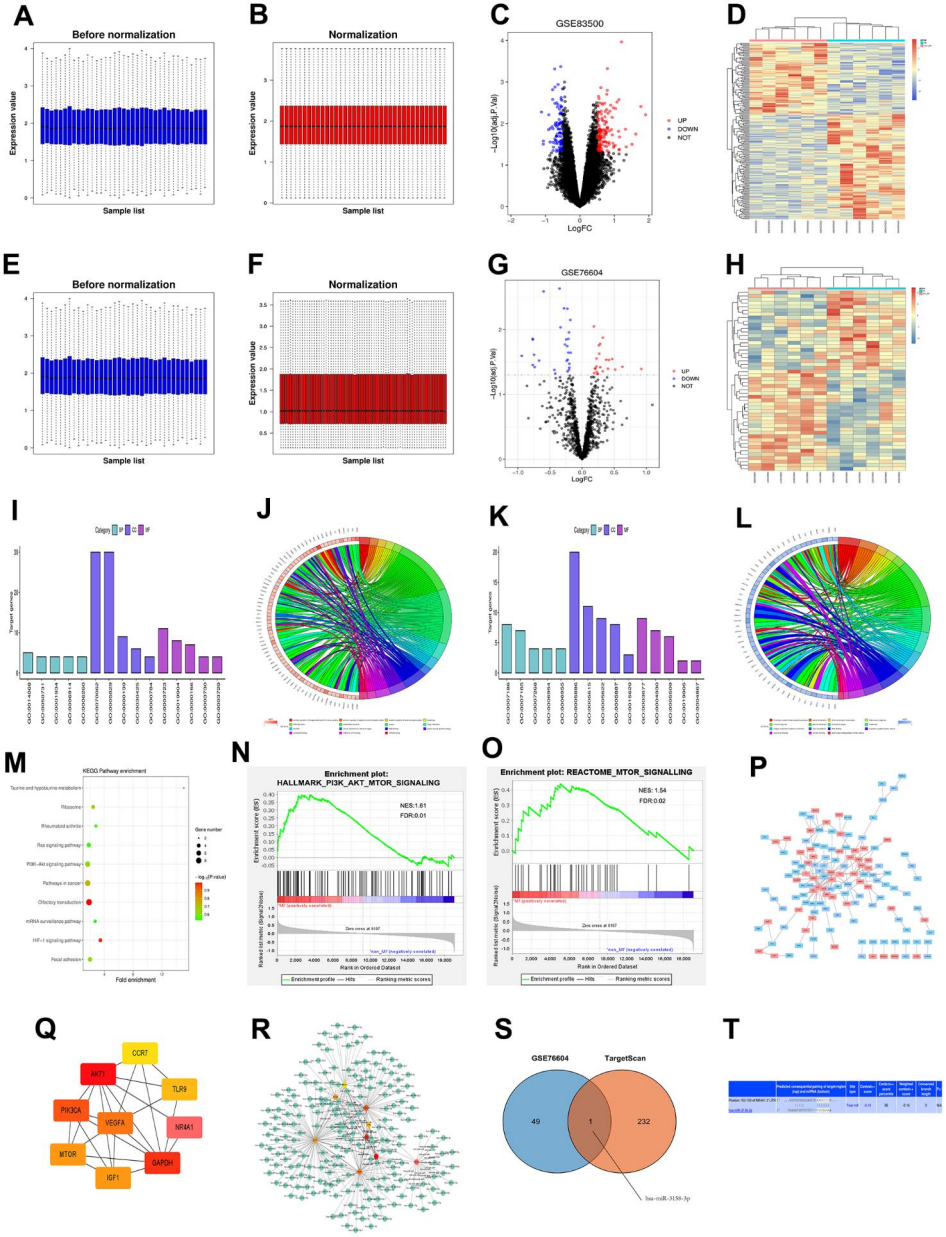
(**A**, **B**) Standardization of GSE83500 dataset, in which blue represents the data before standardization, and red represents the data after standardization. (**C**) DEGs between two groups of samples in the GSE83500 data set, in which red represents up-regulated genes, blue represents down-regulated genes, and black represents genes with no significant difference in expression (/logFC/>1, P<0.05); (**D**) Clustering heat map of top 100 differential genes, in which red represents relatively up-regulated genes, blue represents relatively down-regulated genes, and yellow indicates genes with no significant difference in expression. (**E**, **F**) Standardization of the GSE83500 data set, where blue represents the data before standardization, and red represents the data after standardization. (**G**) DEGs between the two groups in the GSE83500 data set, where red represents up-regulated genes, blue represents down-regulated genes, and black represents genes with no significant difference in expression (/logFC/>1, P<0.05); (**H**) Cluster heat map of top 100 DEGs, where red represents relatively up-regulated genes, blue represents relatively down-regulated genes, and yellow indicates genes with no significant difference in expression. (**I**) Enrichment of the top 15 up-regulated GO terms. (**J**) Enrichment function of up-regulated DEGs. (**K**) Enrichment of the top 15 down-regulated GO terms. (**L**) Enrichment function of down-regulated DEGs. (**M**) KEGG pathway analysis of DEGs. (**N**, **O**) GSEA of the enrichment pathway. (**P**) PPI network analysis diagram of enrichment pathways, where red represents up-regulated genes, and blue represents down-regulated genes. (**Q**) Top 10 target genes in PPI network score. (**R**) Target genes of miRNA predicted using targetScan. (**S**) Venn diagram showing common miRNAs. (**T**) The binding site between mRNAs and miRNAs.

The myocardial infarction-related data set GSE83500 was downloaded from the GEO database, and the data were normalized by quantiles (Figure 1A, 1B). According to the criteria of P<0.05 and /logFC/>1, GSE83500 was screened. The results revealed that there were 248 DEGs in the mRNAs involved in myocardial infarction, of which 150 were up-regulated, and 98 were down-regulated. Then ggplot2 software package was utilized to plot the visualized volcano map of grouped DEGs in GSE83500 in R software (Figure 1C), and the cluster analysis heat map of DEGs was drawn by R software package pheatmap (Figure 1D). The quantile standardization of the miRNA data set GSE76604 was performed using the same method (Figure 1E, 1F). Then DEGs were screened, and their volcano map (Figure 1G) and cluster analysis heat map (Figure 1H) were plotted.

### Bioinformatics analysis results

GO and KEGG enrichment analyses were conducted for DEGs in GSE83500. Besides, the online database tool DAVID (https://david.ncifcrf.gov/) was employed to analyze the DEGs in biological processes to integrate GO terms and create a biological process network of DEGs. Subsequently, R language was utilized to plot the diagrams of up-regulated (Figure 1I, 1J) and down-regulated (Figure 1K, 1L) GO pathways of DEGs. Up-regulated pathways included the positively regulated PI3K signaling pathway, positively regulated nuclear-transcribed mRNA catabolic process, adenosine-independent decay and cell responses to VEGF stimulation, while down-regulated pathways included G protein-coupled receptor signaling pathway, signal transduction, and chemical synaptic transmission, which were enrichment pathways of myocardial infarction. Additionally, the KEGG pathway was analyzed using DEGs, and its diagram was drawn (Figure 1M).

### GSEA results

GSEA results manifested that gene sets were enriched in the PI3K and mTOR signaling pathways (Figure 1N, 1O).

### Results of PPI network analysis of DEGs and screening of target genes

DEGs were imported into the STRING database to obtain the PPI network (Figure 1P). Then the PPI network was imported using Cytoscape, and cytoHubba plug-in was utilized for calculation. Finally, target genes with a score <10 were obtained (Figure 1Q).

### Prediction results of miRNA target genes

The miRNA prediction was carried out for the target genes obtained in section 2.4 using targetScan (Figure 1R). Next, Venn diagrams of these target genes and GSE76604 DEGs were drawn, and the intersection was taken to find out miR-3158, an up-regulated coassociative miRNA (Figure 1S). Finally, the binding site between mRNAs and miRNAs was mapped according to gene prediction results (Figure 1T).

### XST improved left ventricular morphology and function in mice with myocardial infarction

**Figure 2:**
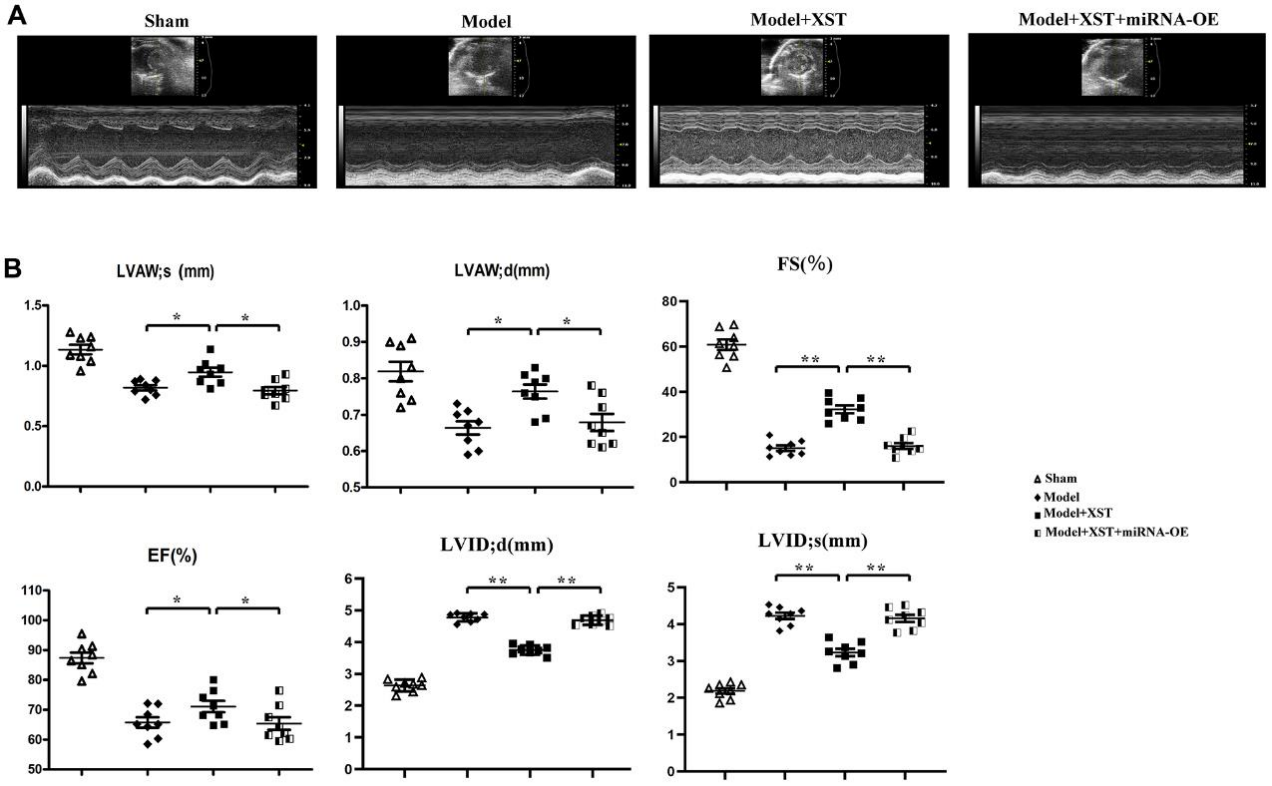
**Changes of left ventricular morphology, structure, and function in mice detected by cardiac ultrasonography.** (**A**) Cardiac ultrasonography images of mice. (**B**) Changes in parameters of mouse cardiac ultrasonography, including LVAWd, LVAWs, LVIDd, LVIDs, FS, and EF.

The ultrasonography results (Figure 2) manifested that compared with those in Control group, the parameters of cardiac ultrasonography including LVAWd, LVAWs, LVIDd, LVIDs, FS, and EF in Sham group exhibited no significant changes. In comparison with those in Model group, LVAW; d, LVAW; s, and EF markedly rose in XST group and decreased in Model + XST + miRNA-OE group.

### XST relieved cardiac fibrosis in mice with myocardial infarction

**Figure 3:**
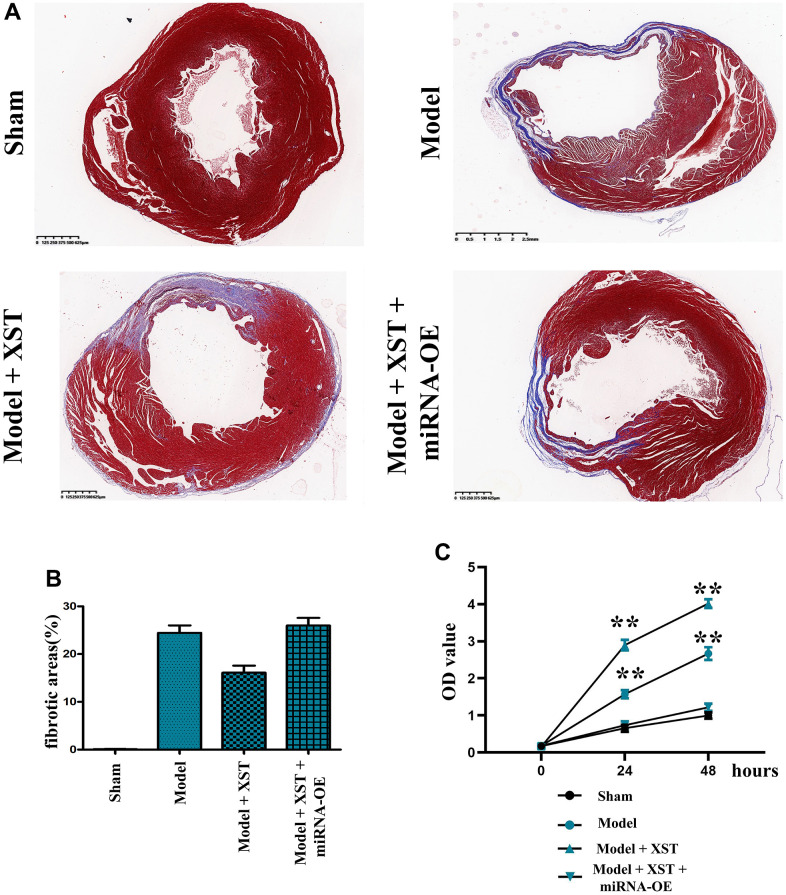
**Changes of cardiac fibrosis in mice and cell proliferation capacity detected via MTT assay.** (**A**) Masson staining of the mouse heart. (**B**) Statistical results of the proportion of cardiac fibrosis areas in mice. (**C**) The OD value detected by MTT.

According to the morphological examination results (Figure 3A, 3B), compared with Control group, Sham group showed fibrosis in few tissues, but Model group displayed obvious tissue fibrosis. Moreover, in contrast to that in Model group, the proportion of fibrotic areas in mice in High-Dose XST group evidently declined (P<0.01), indicating that the cardiac fibrosis of mice with myocardial infarction is notably alleviated.

### XST improved the proliferation and survival of cells in mice with myocardial infarction

**Figure 4:**
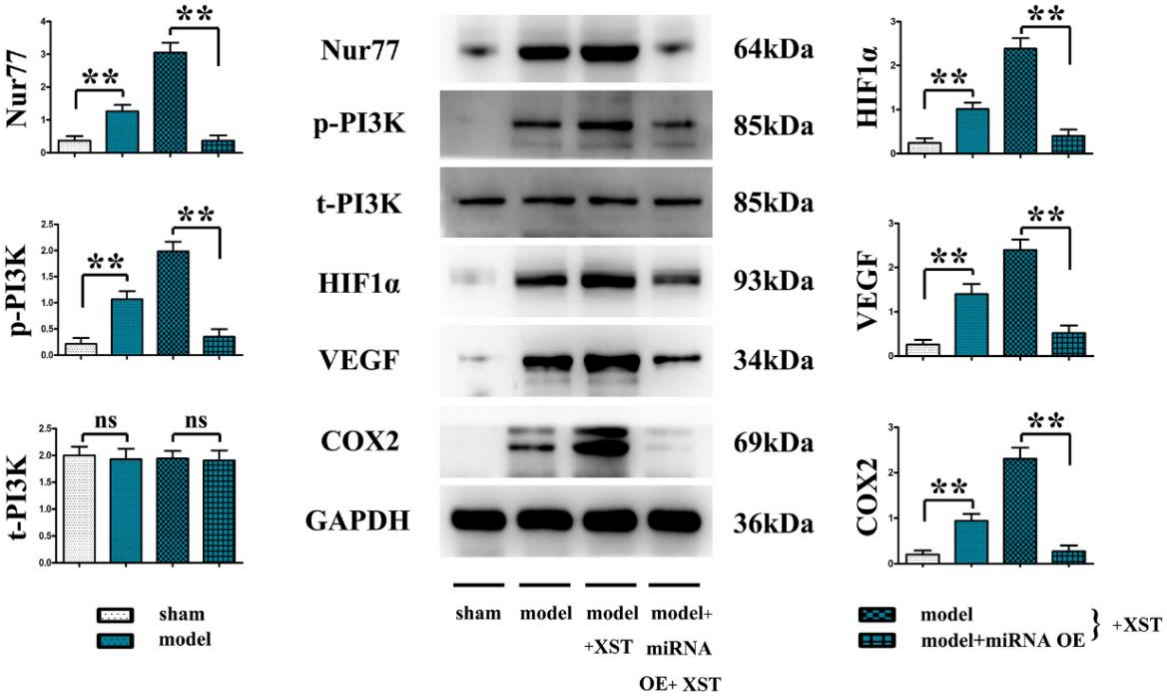
XST facilitates the angiogenesis-associated Nur77, p-PI3K, HIF1α, VEGFs, and COX2 in mice with myocardial infarction by inhibiting the expression of miR-3158-3p in myocardial infarction mice.

The MTT assay results revealed that the OD value in Model + XST group was significantly increased compared with that in Model group (P<0.01), while the OD value in Model + miRNA-OE + XST group was markedly decreased in comparison with Model + XST group (P<0.01) (Figure 4), indicating that XST can increase the survival rate and enhanced the proliferation ability of cells in mice with myocardial infarction cells.

### XST enhanced the tube formation ability in mice with myocardial infarction

**Figure 5:**
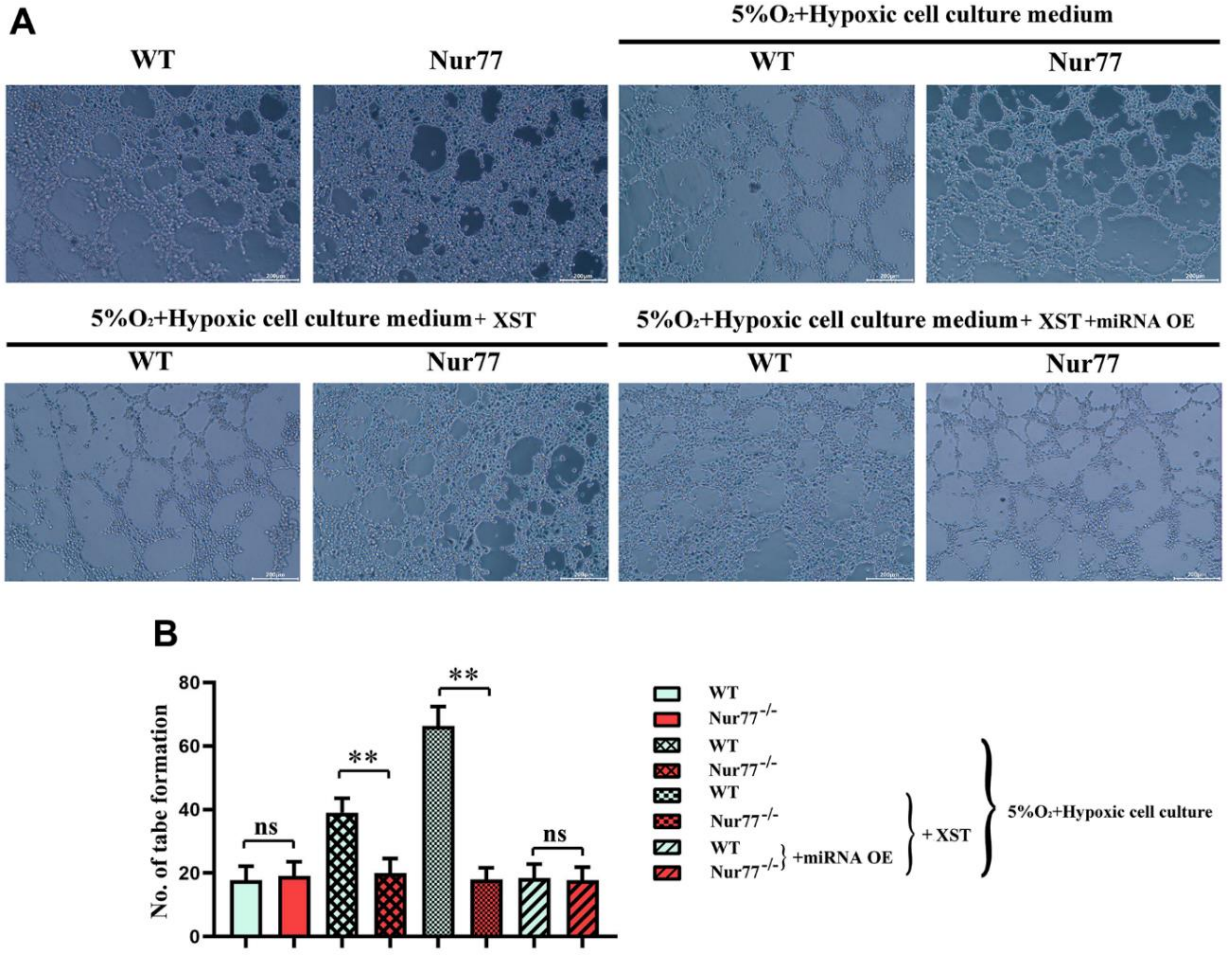
**XST inhibits miR-3158-3p targeting Nur77 and regulates the proliferative capacity of blood vessels.** (**A**) The tube forms a plot of experimental results. (**B**) Tube formation experiment data statistics.

As shown in Figure 5, the tube formation assay results manifested that treatment with XST remarkably enhanced the tube formation ability in WT group (P<0.01), while the addition of miR-3158-3p greatly weakened the tube formation ability. However, no change in the tube formation ability was observed in Nur77^-/-^ mice. The above results suggested that XST affects the tube formation ability by inhibiting miR-3158-3p targeting Nur77 and regulating the proliferative capacity of blood vessels.

### XST facilitated angiogenesis in mice with myocardial infarction by inhibiting the expression of miR-3158-3p

It was found that in contrast to those in Sham group, the protein expressions of Nur77, p-PI3K, HIF-1α, VEGFs, and COX-2 in the heart tissues of mice in Model group were remarkably elevated (P<0.01), and they also evidently rose after XST treatment compared with those in Model group. Moreover, such expressions all declined in Model + miRNA-OE + XST group in comparison with Model + XST group (P<0.01).

### XST inhibited miR-3158-3p targeting Nur77 to facilitate the angiogenesis in cardiomyocytes

The primary cardiomyocytes extracted from Nur77^-/-^ or WT suckling mice were cultured until they reached the logarithmic growth phase. After digestion and suspension, the cell density was adjusted to 6×10^5^/mL.

**Figure 6:**
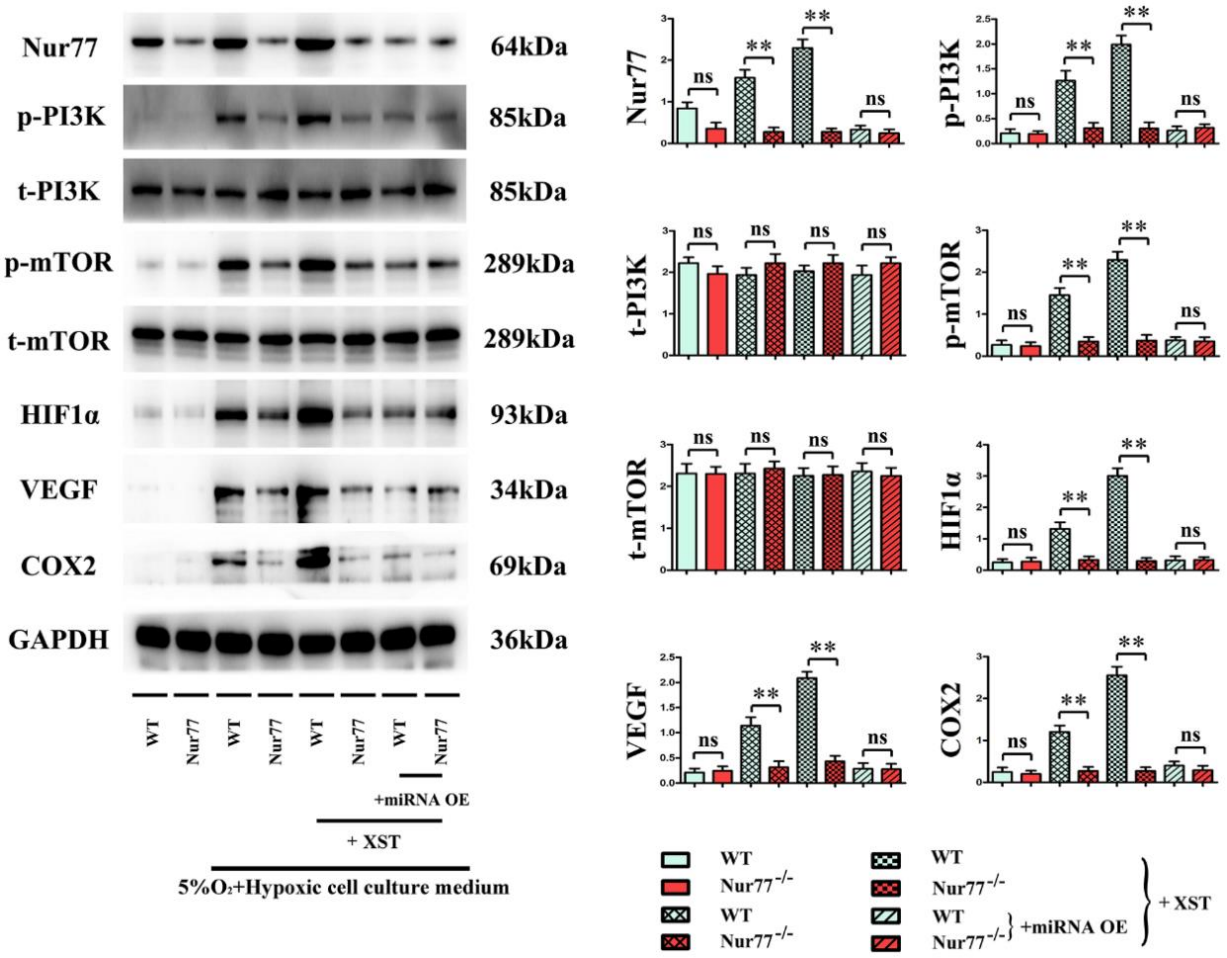
XST inhibits miR-3158-3p targeting Nur77 to facilitate the angiogenesis-associated proteins of cardiomyocytes in *in vitro* experiments.

The cells were then divided into normal group, myocardial infarction group treated with 5% O_2_ and a hypoxic cell culture medium to simulate the myocardial infarction in cells, and miR-3158-3p mimic group treated with XST or miRNA OE. The results manifested that in Model group and XST groups, the protein expression levels of Nur77, p-PI3K, mTOR, HIF-1α, VEGFs, and COX-2 in the heart tissues of Nur77^-/-^ mice were dramatically reduced compared with those of wild-type (WT) mice (P<0.01), implying that the gene deletion of Nur77 inhibits angiogenesis and that XST may play a role by affecting the expression of Nur77. Besides, the above-mentioned protein expressions in the heart tissues of Nur77^-/-^ mice did not change significantly compared with those of WT mice in Model + miRNA-OE + XST group, suggesting that miR-3158-3p can inhibit the expression of Nur77 in a targeted manner (Figure 6).

## DISCUSSION

As lifestyle changes and the living standard experiences improvement, cardiovascular diseases have seriously jeopardized human health and life, among which myocardial infarction has been the “leading killer of health”. It is of great significance for exploring the development, treatment, and prognosis of cardiovascular diseases to observe the changes in cardiac morphological structure, cardiac function, and abnormal wall motion during research on the development process of cardiovascular diseases [15]. Generally, the ventricular wall becomes thinner after the onset of myocardial infarction. The thinned myocardium caused by the partially necrotic myocardium due to previous myocardial infarction results in local ventricular wall thinning [16]. It was found in this study that compared with those in Model group, the LVAWd, LVAWs, and LVPWd in High-Dose XST group all rose significantly, indicating that high-dose XST can reduce LVAWd, LVAWs and LVPWd in mice with myocardial infarction, and reduce the risk of pericardial tamponade caused by ventricular wall rupture and bleeding. In the meantime, the thinning of the ventricular wall may influence the left ventricular EF (%). EF is the main index for evaluating the left ventricular systolic function, which can reflect the myocardial fiber shortening function of the left ventricle [17]. The results also demonstrated that the EF of the left ventricle of mice in Model group was remarkably raised after high-dose XST treatment, further suggesting that high-dose XST can relieve cardiac hypofunction after myocardial infarction in mice. Furthermore, the therapeutic effect of XST on mice with myocardial infarction was examined through morphological observation. The results revealed that after XST treatment, myocardial fibrosis in mice with myocardial infarction was dramatically attenuated.

XST has been shown to protect the ischemic myocardium, resist myocardial ischemia, prevent reperfusion injury, dilate coronary arteries, increase coronary blood flow, improve myocardial metabolism, and up-regulate the expression of the VEGF signaling pathway, thus promoting angiogenesis [5]. Hence, angiogenesis-related proteins in mouse heart tissues were further detected. It was found that the protein expressions of HIF-1α, VEGFs, and COX-2 were increased with the marked increases of Nur77 and p-PI3K protein expressions in the heart of myocardial infarction mice. These results suggested that the process of angiogenesis can be initiated after myocardial ischemia, which increases the new vessels of the ischemic myocardium to raise the blood supply, and that ischemia and hypoxia can trigger the expression of these proteins. In the process of angiogenesis, endothelial cells on the inner wall of blood vessels react to growth signals (such as alkalinity) to form bFGFs, VEGFs, cytokines, or nitric oxides, thus proliferating and migrating to form new blood vessels [18]. With respect to pathology, angiogenesis can facilitate the healing of inflammation and abnormal wounds, but it also can speed up the development and progression of vascular diseases and tumors [19]. Besides, the experimental results also revealed that compared with Model group, XST groups exhibited notably increased expressions of the above-mentioned genes in the heart. A study demonstrated that the PI3K signaling pathway, the key to signal transduction in normal cells, exerts a vital effect on the occurrence and development of tumors [20]. In addition, it also plays an indispensable role in functions such as cell metabolism, migration, proliferation, angiogenesis, adhesion and invasion [21], illustrating that XST’s promoting effect on angiogenesis after myocardial infarction in mice is probably associated with the activation of the PI3K signaling pathway. Moreover, in comparison with those in Model + XST group, the protein expressions of Nur77, p-PI3K, HIF-1α, VEGFs, and COX-2 in mouse heart tissues all declined in Model + miRNA-OE + XST group (P<0.01), suggesting that miR-3158-3p inhibits the expression of Nur77, p-PI3K and angiogenesis-related proteins. The above results were consistent with the results of bioinformatics analysis, that is, miR-3158-3p was lowly expressed, whereas Nur77 was highly expressed in mice with myocardial infarction.

Hence, Nur77^-/-^ mice were used to further determine whether XST facilitates angiogenesis in mice with myocardial infarction by suppressing miR-3158-3p targeting Nur77. It has been revealed that the deficiency of Nur77 promotes endothelial-interstitial transformation, thus exacerbating myocardial fibrosis after myocardial infarction [22, 23]. The results of this study manifested that in Model group and XST groups, the protein expression levels of Nur77, p-PI3K, mTOR, HIF-1α, VEGFs, and COX-2 in the heart tissues of Nur77^-/-^ mice were dramatically reduced compared with those of WT mice (P<0.01), reflecting that the gene deletion of Nur77 inhibits angiogenesis, which is consistent with previous study results, and that XST may play a role by affecting the expression of Nur77. Besides, the above-mentioned protein expressions in the heart tissues of Nur77^-/-^ mice did not change significantly compared with those of WT mice in Model + miRNA-OE + XST group, suggesting that miR-3158-3p is able to inhibit the expression of Nur77 in a targeted way. In a word, XST inhibits miR-3158-3p targeting Nur77 to facilitate myocardial angiogenesis in mice with myocardial infarction.

## References

[1] Jaffe EA. Culture of human endothelial cells. Transplant Proc. 1980; 12:49–53. 6254221

[2] Ferrara N. Role of vascular endothelial growth factor in physiologic and pathologic angiogenesis: therapeutic implications. Semin Oncol. 2002; 29:10–4. 10.1053/sonc.2002.37264 12516033

[3] Phng LK, Gerhardt H. Angiogenesis: a team effort coordinated by notch. Dev Cell. 2009; 16:196–208. 10.1016/j.devcel.2009.01.015 19217422

[4] Mohr T, Haudek-Prinz V, Slany A, Grillari J, Micksche M, Gerner C. Proteome profiling in IL-1β and VEGF-activated human umbilical vein endothelial cells delineates the interlink between inflammation and angiogenesis. PLoS One. 2017; 12:e0179065. 10.1371/journal.pone.0179065 28617818 PMC5472280

[5] Telfer CM, Dow RC, Fink G, Booth NA. The ginseng preparation Xuesaitong up-regulates plasminogen activator expression in human endothelial cells. 1998.

[6] Xue R, Zhai R, Xie L, Zheng Z, Jian G, Chen T, Su J, Gao C, Wang N, Yang X, Xu Y, Gui D. Xuesaitong Protects Podocytes from Apoptosis in Diabetic Rats through Modulating PTEN-PDK1-Akt-mTOR Pathway. J Diabetes Res. 2020; 2020:9309768. 10.1155/2020/9309768 32051833 PMC6995497

[7] Ma X, Chen Y, Jiang S, Zhao X. A Bioassay-Based Approach for the Batch-To-Batch Consistency Evaluation of Xuesaitong Injection on a Zebrafish Thrombosis Model. Front Pharmacol. 2021; 12:623533. 10.3389/fphar.2021.623533 33762944 PMC7982889

[8] Zhou D, Cen K, Liu W, Liu F, Liu R, Sun Y, Zhao Y, Chang J, Zhu L. Xuesaitong exerts long-term neuroprotection for stroke recovery by inhibiting the ROCKII pathway, *in vitro* and *in vivo*. J Ethnopharmacol. 2021; 272:113943. 10.1016/j.jep.2021.113943 33617967

[9] Duan X, Zhang D, Wang K, Wu J, Zhang X, Zhang B, Gao X. Comparative study of xuesaitong injection and compound salvia miltiorrhizae injection in the treatment of acute cerebral infarction: a meta-analysis. J Int Med Res. 2019; 47:5375–88. 10.1177/0300060519879292 31594441 PMC6862920

[10] Wei JW, Heeley EL, Wang JG, Huang Y, Wong LK, Li Z, Heritier S, Arima H, Anderson CS, and ChinaQUEST Investigators. Comparison of recovery patterns and prognostic indicators for ischemic and hemorrhagic stroke in China: the ChinaQUEST (QUality Evaluation of Stroke Care and Treatment) Registry study. Stroke. 2010; 41:1877–83. 10.1161/STROKEAHA.110.586909 20651267

[11] Ruan L, Wang B, ZhuGe Q, Jin K. Coupling of neurogenesis and angiogenesis after ischemic stroke. Brain Res. 2015; 1623:166–73. 10.1016/j.brainres.2015.02.042 25736182 PMC4552615

[12] Boro A, Bauer D, Born W, Fuchs B. Plasma levels of miRNA-155 as a powerful diagnostic marker for dedifferentiated liposarcoma. Am J Cancer Res. 2016; 6:544–2. 10.1158/1538-7445.AM2016-1941 27186423 PMC4859679

[13] Urbich C, Kuehbacher A, Dimmeler S. Role of microRNAs in vascular diseases, inflammation, and angiogenesis. Cardiovasc Res. 2008; 79:581–8. 10.1093/cvr/cvn156 18550634

[14] Ueda S, Yamagishi S, Matsui T, Jinnouchi Y, Imaizumi T. Administration of pigment epithelium-derived factor inhibits left ventricular remodeling and improves cardiac function in rats with acute myocardial infarction. Am J Pathol. 2011; 178:591–8. 10.1016/j.ajpath.2010.10.018 21281791 PMC3278887

[15] Nagueh SF, Middleton KJ, Kopelen HA, Zoghbi WA, Quiñones MA. Doppler tissue imaging: a noninvasive technique for evaluation of left ventricular relaxation and estimation of filling pressures. J Am Coll Cardiol. 1997; 30:1527–33. 10.1016/s0735-1097(97)00344-6 9362412

[16] Montalto C, Kotronias RA, Marin F, Terentes-Printzios D, Shanmuganathan M, Emfietzoglou M, Scalamera R, Porto I, Langrish J, Lucking A, Choudhury R, Kharbanda R, Channon KM, et al, and Oxford Acute Myocardial Infarction (OxAMI) Study. Pre-procedural ATI score (age-thrombus burden-index of microcirculatory resistance) predicts long-term clinical outcomes in patients with ST elevation myocardial infarction treated with primary percutaneous coronary intervention. Int J Cardiol. 2021; 339:1–6. 10.1016/j.ijcard.2021.07.040 34311009

[17] Kim HR, Jeong DS, Kwon HJ, Park SJ, Park KM, Kim JS, On YK. Total thoracoscopic ablation in patients with atrial fibrillation and left ventricular dysfunction. JTCVS Tech. 2021; 8:60–6. 10.1016/j.xjtc.2021.04.006 34401814 PMC8350785

[18] Goto F, Goto K, Weindel K, Folkman J. Synergistic effects of vascular endothelial growth factor and basic fibroblast growth factor on the proliferation and cord formation of bovine capillary endothelial cells within collagen gels. Lab Invest. 1993; 69:508–17. 8246443

[19] Aird WC. The role of the endothelium in severe sepsis and multiple organ dysfunction syndrome. Blood. 2003; 101:3765–77. 10.1182/blood-2002-06-1887 12543869

[20] Bader AG, Kang S, Zhao L, Vogt PK. Oncogenic PI3K deregulates transcription and translation. Nat Rev Cancer. 2005; 5:921–9. 10.1038/nrc1753 16341083

[21] Cain RJ, Vanhaesebroeck B, Ridley AJ. The PI3K p110alpha isoform regulates endothelial adherens junctions via Pyk2 and Rac1. J Cell Biol. 2010; 188:863–76. 10.1083/jcb.200907135 20308428 PMC2845076

[22] Nur77 deficiency exacerbates cardiac fibrosis after myocardial infarction through promoting endothelial to mesenchymal transition. European Heart Journal 41. 2020. 10.1093/ehjci/ehaa946.3738 32542822

[23] Niu G, Ye T, Qin L, Bourbon PM, Chang C, Zhao S, Li Y, Zhou L, Cui P, Rabinovitz I, Mercurio AM, Zhao D, Zeng H. Orphan nuclear receptor TR3/Nur77 improves wound healing by upregulating the expression of integrin β4. FASEB J. 2015; 29:131–40. 10.1096/fj.14-257550 25326539 PMC4285545

