# Correction for: Xuesaitong promotes myocardial angiogenesis in myocardial infarction mice by inhibiting MiR-3158-3p targeting Nur77

**DOI:** 10.18632/aging.205576

**Published:** 2024-01-30

**Authors:** Jiangquan Liao, Mingjing Shao, Yan Wang, Peng Yang, Dongliang Fu, Mengru Liu, Tong Gao, Kangkang Wei, Xianlun Li, Jinhang Du

**Affiliations:** 1 National Integrated Traditional and Western Medicine Center for Cardiovascular Disease, China–Japan Friendship Hospital, Beijing, China; 2 Department of Cardiology, Beijing Tsinghua Changgung Hospital, Medical Center, Tsinghua University, Beijing, China; 3 Graduate School, Beijing University of Chinese Medicine, Beijing, China

**Keywords:** Xuesaitong, miR-3158-3p, myocardial infarction, angiogenesis

**This article has been corrected:** To correct a miscommunication with the journal, the authors have changed the order in which they are listed. The first author is now Yan Wang, who contributed equally to this study with Jiangquan Liao and also provided funding to the study. Correspondingly, the funding information has been updated and now lists support from National High Level Hospital Clinical Research Funding, Elite Medical Professionals Project of China-Japan Friendship Hospital (NO.ZRJY2023-QM24).

The corrected **authors order** and **funding section** are shown below.

Yan Wang^1,*^, Jiangquan Liao^1,*^, Mingjing Shao^1^, Peng Yang^1^, Dongliang Fu^1^, Mengru Liu^1^, Tong Gao^2^, Kangkang Wei^3^, Xianlun Li^1^, Jinhang Du^1^

^1^National Integrated Traditional and Western Medicine Center for Cardiovascular Disease, China–Japan Friendship Hospital, Beijing, China

^2^Department of Cardiology, Beijing Tsinghua Changgung Hospital, Medical Center, Tsinghua University, Beijing, China

^3^Graduate School, Beijing University of Chinese Medicine, Beijing, China

*Equal contribution

